# Cardiometabolic Risk Factors Associated with Magnesium and Vitamin D Nutrients during Pregnancy—A Narrative Review

**DOI:** 10.3390/nu16162630

**Published:** 2024-08-09

**Authors:** Maisha Naowar, Darby Dickton, Jimi Francis

**Affiliations:** 1 Department of Public Health, College of Health, Community, and Policy, University of Texas at San Antonio, San Antonio, TX 78249, USA; maisha.naowar@my.utsa.edu; 2 Foundation for Maternal, Infant, and Lactation Knowledge, San Antonio, TX 78249, USA; ddickton@foundation4milk.org; 3 Department of Kinesiology, College of Health, Community, and Policy, University of Texas at San Antonio, San Antonio, TX 78249, USA

**Keywords:** gestational diabetes, gestational hypertension, cardiometabolic changes, vitamin D, magnesium

## Abstract

This narrative review comprehensively explores the cardiometabolic implications of two vital nutrients, magnesium and vitamin D, during gestation. Magnesium, a key regulator of vascular tone, glucose metabolism, and insulin sensitivity, plays a crucial role in mitigating gestational hypertension and diabetes, a point this review underscores. Conversely, vitamin D, critical for immune response and calcium level maintenance, is linked to gestational diabetes and hypertensive disorders of pregnancy. The authors aim to enhance comprehension of the complex interaction between these nutrients and cardiometabolic function in pregnancy, knowledge that is pivotal for optimizing maternal–fetal outcomes. The mother’s health during pregnancy significantly influences the long-term development of the fetus. Recognizing the impact of these nutrient deficiencies on the physiology of cardiometabolic cycles underscores the importance of adequate nutritional support during pregnancy. It also emphasizes the pressing need for future research and targeted interventions to alleviate the burden of pregnancy complications, highlighting the crucial role of healthcare professionals, researchers, and policy makers in obstetrics and gynecology in this endeavor.

## 1. Introduction

Maternal health during pregnancy is paramount for the well-being of both the mother and the developing fetus. Vitamin D and magnesium deficiencies have attracted attention due to their potential impact on cardiometabolic health [1,2]. This review explores the physiological roles of magnesium and vitamin D during pregnancy. It delves into the existing literature on how deficiencies in these essential nutrients may contribute to cardiometabolic risks.

Hypertensive disorders of pregnancy (HDPs) are one of the most frequent cardiometabolic complications during pregnancy [3]. They encompass chronic hypertension, gestational hypertension, preeclampsia, and eclampsia, affecting up to 16% of pregnancies and posing a significant risk for the mother [4]. HDPs are associated with increased maternal morbidity and mortality.

This narrative review synthesizes findings from observational and interventional studies, providing a comprehensive understanding of how magnesium and vitamin D deficiencies intersect with cardiometabolic outcomes during pregnancy. The topics discussed encompass blood pressure regulation, glucose metabolism, lipid profiles, and inflammatory markers. The review also addresses the effect of these deficiencies on infant development and cardiovascular health as an adult.

## 2. Pathophysiology of HDPs

HDPs impact approximately 35% of women with gestational hypertension [5], and of those with chronic hypertension, approximately 25% are impacted [6]. Pregnancy-induced hypertension begins with an initial event that triggers dysfunction in the maternal vascular endothelium. This endothelial malfunction increases the sensitivity of blood vessels to angiotensin II as a response to increased production of endothelin and thromboxane. It decreases the production of the vasodilators nitric oxide and prostacyclin [7]. Pregnancy is characterized by significant hemodynamic adaptations to support fetal growth. These adaptations include increased blood volume, cardiac output, and peripheral vascular resistance. However, these adaptations may not occur adequately in some women, leading to increased vascular resistance and elevated blood pressure. While more research is needed to establish definitive guidelines, evidence suggests inadequate magnesium and vitamin D intake. Vitamin D may help to combat hypertensive disorders of pregnancy by supporting vascular health, reducing inflammation, and promoting overall maternal and fetal well-being.

## 3. Overview of the Prevalence of Magnesium and Vitamin D Deficiencies during Pregnancy

Many adults do not meet the recommendations for magnesium intake [8]. Magnesium deficiency is a widespread concern, affecting a significant portion of the global population due to dietary and lifestyle factors. Many people struggle to obtain adequate magnesium from their diet because modern agricultural practices have depleted soil magnesium levels, reducing its magnesium content [9]. Additionally, processed foods, which are often low in magnesium, dominate many diets [10]. These challenges are further compounded by the high intake of substances that deplete magnesium, such as alcohol, caffeine, and certain medications [11]. As a result, despite magnesium’s critical role in over 300 biochemical processes, including muscle function and bone health, achieving sufficient intake through diet alone can be difficult. While magnesium can be absorbed through the skin, it is not a significant source of magnesium for most people. Women between the ages of 15 to 49 years of age commonly have magnesium deficiency. At the highest risk are pregnant women. Magnesium deficiency in pregnancy impacts maternal health and affects the infant’s health. The consequences of magnesium deficiency can be far-reaching, affecting the mother, the developing fetus, and the child’s future health. During pregnancy, low levels of magnesium may interfere with fetal growth and increase the risk of preterm birth. Chronically low levels of magnesium can result in uterine hyperexcitability [12], which can be worsened by maternal anxiety. A secondary exploration of data from four extensive observational studies evaluated low magnesium during pregnancy, including women with HRC (hormone-related condition). The dataset included 983 pregnant participants, with 9444 women presenting with HRC. The prevalence of low serum magnesium of <0.8 mmol/L was higher in the pregnant group at 78% compared to 55% in the HRC group. These results emphasize the importance of routine screening since the risk factors are generally nonspecific [13].

Vitamin D deficiency is a pressing global issue, affecting a substantial portion of the population across various age groups and geographic locations [14]. The prevalence is notably high due to several factors, including limited sun exposure, particularly in northern latitudes or during winter months, and low dietary intake of vitamin D-rich foods [15]. Many individuals do not consume enough vitamin D through diet alone, as it is found in relatively few foods such as fatty fish, liver, and fortified products [16]. Furthermore, modern lifestyles that involve long hours indoors and the use of sunscreen, which reduces vitamin D synthesis from sunlight, exacerbate the problem. Vitamin D deficiency varies globally, influenced by geographical location and cultural practices. The amount of sunshine exposure due to latitude and season and food routines can lead to lower serum vitamin D. A recent review of pregnant women and neonates described vitamin D deficiency rates as 54% and 75%, respectively [17]. This deficiency is a significant worldwide concern during pregnancy, impacting maternal and fetal health outcomes. Vitamin D, also called 25-hydroxyvitamin D or calcitriol in serum, is transformed into its active form, 1,25-dihydroxy vitamin D, by the enzyme CYP27B1 [18]. This enzyme is expressed in the placenta but is mainly found in the kidney. Pregnancy is a unique physiological condition as the placenta is crucial in metabolizing this vitamin [19]. Infants of mothers with low vitamin D levels also have an increased risk of deficiency. A worldwide review reported the prevalence of 25(OH)D deficiency (<50 nmol/L) in pregnant women, with the highest level of deficiency in Southeast Asia at 87%, followed by the Western Pacific at 83%, the Americas at 64%, Europe at 57%, and Eastern Mediterranean at 46% based on WHO regions [20]. A literature review based on South Asian countries evaluated primary research on pregnant women and reported a prevalence of vitamin D deficiency among females of 76% [21,22]. The international guidelines for screening and supplementing vitamin D during gestation vary widely. However, there is a consensus on a substantial knowledge gap, necessitating clear proof of the efficacy, optimal dosage, and scheduling of vitamin D supplementation among pregnant women.

## 4. In-Depth Exploration of the Physiological Roles of Magnesium during Gestation

Magnesium is critical for various physiological processes, including energy metabolism, muscle function, and DNA synthesis [23]. It regulates blood pressure, glucose metabolism, and fetal bone development during pregnancy [24].

Research indicates that magnesium deficiency during pregnancy is common. Adverse maternal and fetal outcomes are associated with this deficiency [25]. It is theorized that magnesium deficiency is correlated with gestational hypertension [26]. Studies also indicate that inadequate magnesium levels may contribute to preeclampsia [27] and increase the risk of gestational diabetes [28]. This section reviews the existing evidence on the influence of magnesium deficiency on cardiometabolic health during pregnancy.

### 4.1. Impact of Magnesium Deficiency on HDPs

HDPs are substantial pregnancy complications that pose risks to the mother and fetus. Practitioners have known the importance of magnesium for cardiac function for many years [29,30]. The importance of magnesium becomes even more pronounced during pregnancy. Many cardiometabolic functions are modulated by magnesium, such as intracardiac conduction and neuronal excitation. Magnesium is pivotal for myocardial function due to the regulation of ion transporters [31]. Magnesium serves as a calcium channel blocker, assisting in balancing calcium levels within cardiac cells [2]. This action prevents excessive calcium influx, supporting normal heart muscle contractility and rhythm [32]. Adequate magnesium levels during pregnancy are necessary for ensuring the proper performance of the maternal cardiovascular system, which is crucial for maternal well-being and the health of the developing infant in utero [33].

During gestation, fluctuations in blood pressure are common, and magnesium helps support vascular tone and elasticity [34]. It acts as a vasodilator [35], promoting the relaxation of blood vessels, which can contribute to managing hypertension. In preeclampsia, inadequate magnesium levels may contribute to vasoconstriction and increased blood pressure [36].

Another study by Standley et al. (1997) [37] showed a pattern of decreasing magnesium, both total and ionized levels, as pregnancy progresses. This study demonstrated an imbalance in magnesium in those women who developed HDP. In patients with acute myocardial infarction, several clinical studies have indicated a decrease in arrhythmias and a reduction in mortality following the administration of 4 to 6 g intravenous magnesium, resulting in a mean decrease of 4.18 mm Hg systolic and 2.27 mm Hg in diastolic pressures [38]. According to research from 2015, magnesium stabilizes electrolyte concentrations. It may help prevent arrhythmias by blocking calcium, restricting the amount that enters the heart cells, and allowing the heart to beat more slowly [9].

This mineral has a therapeutic role in treating pre-eclampsia [36]. A Cochrane meta-analysis has confirmed that magnesium sulfate treatment can reduce the risk of advancement to eclampsia by >50% in preeclamptic women and may also lower the risk of maternal death [39].

**Figure 1:**
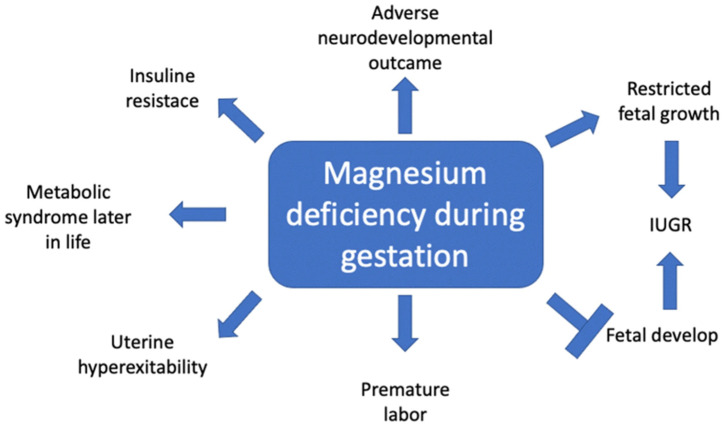
Adverse effects of magnesium deficiency during pregnancy. IUGR = intrauterine growth restriction. Figure adapted from [23].

These complex interactions are shown in Figure 1.

### 4.2. Association between Magnesium Deficiency and Glucose Tolerance

The definition of gestational diabetes mellitus (GDM) is a metabolic disruption leading to increased blood glucose that is commonly detected in the second trimester of pregnancy [40,41]. The body cannot produce adequate insulin to meet increased needs during gestation, leading to elevated blood glucose levels and an amplified risk of complications for mother and infant in utero. GDM affects more than 7% of women in the perinatal period, leading to more than 200,000 incidents annually in the United States alone [42]. Increasing scientific evidence supports a link between the incidence of GDM and magnesium deficiency. A study showed that the body’s sensitivity to insulin was improved and that the incidence of GDM decreased when magnesium supplementation was used for pregnant women at a high risk of diabetes [43].

Magnesium has a part in glucose uptake and insulin sensitivity in glucose utilization [25]. Magnesium is a cofactor for enzymes involved in insulin action, influencing the body’s ability to use glucose [44]. Adequate magnesium levels improve insulin sensitivity, helping support normal blood glucose levels [11]. When insulin resistance increases during pregnancy [45], ensuring sufficient magnesium intake is crucial for managing glucose metabolism. Its deficiency may contribute to gestational diabetes mellitus (GDM) and other metabolic complications, highlighting the importance of maintaining the best magnesium levels for maternal metabolic health [28]. Pregnant individuals are encouraged to meet their dietary magnesium requirements through a balanced and varied diet. Recommended Daily Allowance recommendations are shown in Table 1.

During gestation, the body exhibits resistance to insulin due to the placental secretion of hormones. This resistance likely ensures fetal receipt of sufficient glucose by shifting maternal metabolism from predominantly carbohydrate utilization to lipids [46]. Magnesium supplementation improves glucose uptake in individuals with diabetes and improves insulin sensitivity markers for those with a heightened risk of developing elevated blood glucose. Moreover, a six-week magnesium supplementation regimen of 250 mg/day magnesium oxide in women with GDM has been demonstrated to impact gene expression and inflammatory markers associated with energy metabolism affected by GDM, specifically downregulating tumor necrosis factor and interleukin 8 (IL-8) while upregulating growth factor beta (TGF-β) [47].

### 4.3. Impact of Magnesium on Fetal Development

Magnesium is critical in fetal bone development and mineralization. Magnesium forms and supports fetal skeleton development, with calcium and phosphorus supporting fetal skeleton development [5,47]. The mineral facilitates the synthesis of DNA and RNA, which is critical for cellular development and differentiation in growing bones [48]. Magnesium deficiency during pregnancy may compromise fetal bone mineralization and increase the risk of skeletal abnormalities [47]. Ensuring an adequate supply of magnesium is crucial for supporting the growing needs of the developing fetus, particularly concerning bone formation [49].

Magnesium plays a multifaceted role during pregnancy, changing cardiac function, blood pressure regulation, glucose metabolism, and fetal bone development. Maintaining optimal magnesium levels is essential for supporting maternal cardiovascular health, preventing hypertensive disorders, managing glucose metabolism, and ensuring proper skeletal growth in the developing fetus.

## 5. In-Depth Exploration of the Functional Roles of Vitamin D during Gestation

In 1922, the term “vitamin D” began to designate the chemical compound needed to promote calcium absorption and metabolism in the body [50]. Emerging research has highlighted the significance of vitamin D in supporting glucose homeostasis [51], regulating insulin sensitivity [52], and influencing blood pressure [53], all of which are pivotal aspects of maternal and fetal health during pregnancy.

Both bone formation and calcium homeostasis depend on the presence of vitamin D [47]. It also affects immune function [53] and has been implicated in insulin sensitivity [54]. The lack of vitamin D has become a worldwide health concern [55]. Pregnant women are particularly vulnerable to deficiency [56]. The review examines the role of vitamin D in maintaining glucose homeostasis, regulating insulin sensitivity, and influencing blood pressure during pregnancy. The research literature indicates a link between vitamin D deficiency and gestational diabetes, hypertensive disorders, and other cardiometabolic risks.

### 5.1. Examination of the Relationship of Vitamin D Deficiency and HDP

The renin–angiotensin system (RAS) is a key regulator of blood pressure. It needs vitamin D, which inhibits the release of renin, an enzyme involved in blood pressure regulation. The presence of vitamin D modulates the RAS, supporting normal blood pressure. This becomes particularly relevant during pregnancy when fluctuations in blood pressure can have significant implications for maternal and fetal health [56].

Under the umbrella of HDP, preeclampsia is defined as high blood pressure accompanied by protein in the urine, usually seen after the 20th week of gestation [57,58,59]. Vitamin D is crucial for endothelial function and vascular health, and its deficiency may contribute to impaired vascular relaxation and increased systemic vascular resistance, which are characteristic features of HDP. Vitamin D insufficiency increases arterial stiffness and endothelial dysfunction in blood vessels, inducing atherogenesis with a critical role in controlling blood pressure. In contrast to insufficiency, vitamin D deficiency may contribute to inflammatory response and endothelial cell dysfunction. Arterial stiffness is an essential arterial phenotype and an excellent cardiovascular morbidity and mortality indicator [60]. Malfunctions in the blood vessel endothelium and stiffening of arteries are associated with vitamin D insufficiency [61].

Vitamin D contributes to vascular health, influencing the endothelium and smooth muscle cells. Vitamin D, at adequate levels, is correlated with better endothelial function, vasodilation, and overall vascular health. These effects influence the control of blood pressure and may help mitigate HDP. Vitamin D deficiency is associated with many metabolic and cardiovascular diseases (CVDs). As a result, supplementary vitamin D is employed in treating and preventing these conditions. Vitamin D protects blood vessels [62], while its insufficiency poses the risk of endothelial dysfunction. Numerous reports indicate an association between a higher risk of HDP and a lower level of maternal vitamin D. Low maternal vitamin D levels in the second trimester were related to a higher risk of HDPs [63].

### 5.2. Vitamin D Functions in Glucose Homeostasis and Insulin Sensitivity during Gestation

Vitamin D regulates insulin sensitivity through various cellular signaling pathways. It modulates the expression of insulin receptor genes and improves insulin signaling, contributing to improved glucose uptake by cells. Research suggests adequate vitamin D levels may mitigate insulin resistance, reducing the risk of GDM and other metabolic complications during pregnancy [64].

Numerous studies have explored how vitamin D levels influence maternal glucose metabolism and insulin sensitivity, seeking to understand its significance for the health of both mothers and fetal development. A circulating level of 25-hydroxyvitamin D higher than 30 ng/mL is needed to maintain healthy vitamin D status. Existing evidence suggests that serum 25-hydroxyvitamin D of 40–60 ng/mL (100–150 nmol) is needed during pregnancy. This serum level can be achieved with a daily intake of 4000 IU vitamin D3 [65].

GDM is associated with placental hormones that lead to ineffective use of insulin. The sensitivity to insulin typically decreases before the third trimester because of the action of human placental lactogen (HPL), progesterone, and human placental growth hormone (HPGH), which safeguard a constant supply of nutrients to the developing infant in utero [66,67]. As the placenta enlarges, it produces increased amounts of these hormones, heightening the likelihood of insulin resistance. Ordinarily, the pancreas can augment insulin production to counteract this resistance. However, when insulin production does not compensate for placental hormones’ impact, gestational diabetes ensues. These hormones are HPGH, progesterone, and HPL. According to the American Diabetic Association, up to 10% of pregnancies are impacted by GDM each year in the United States. When GDM is untreated or ineffectively treated, the condition leads to negative health consequences for the mother and infant. Maternal vitamin D can exacerbate the consequences of GDM, highlighting its potential implications for both immediate and continuing health implications for the mother and infant [68]. One systemic review and meta-analysis study showed that a mother’s vitamin D status correlates with GDM [69].

Vitamin D plays a role in the regulation of glucose homeostasis by promoting the secretion of insulin from pancreatic B cells [70,71]. Consequently, addressing low levels of vitamin D may improve glucose management and beneficial outcomes in managing complications associated with Type 2 diabetes [72]. Vitamin D receptors are in pancreatic beta cells and skeletal muscles, suggesting their direct roles in insulin secretion and sensitivity [50].

Adequate vitamin D levels may thus play a critical role in preserving glucose balance and insulin sensitivity throughout pregnancy, potentially lowering the likelihood of gestational diabetes mellitus (GDM) and its associated complications.

Vitamin D regulates insulin sensitivity [73], a process critical for glucose homeostasis. Changes in insulin resistance may lead to GDM during gestation [74], which is associated with associated with harmful mother/infant consequences [75]. Vitamin D interacts with insulin receptors and contributes to the proper functioning of insulin-sensitive tissues, helping keep glucose levels within a healthy range [76].

Vitamin D also influences pancreatic beta-cell function, which is responsible for the production of insulin. Proper beta-cell function is essential for the appropriate insulin release in response to glucose levels. This function may be inhibited by low vitamin D levels, which contribute to disturbances in glucose metabolism.

Vitamin D supports glucose homeostasis, regulates insulin sensitivity, and influences blood pressure during pregnancy, which are complex and interconnected. Adequate vitamin D levels are essential for optimal maternal metabolic health. They may contribute to a reduced risk of GDM and HDP. However, the precise locations in metabolic pathways impacted by vitamin D during pregnancy are poorly understood and call for further research. Ensuring sufficient vitamin D intake through diet, sunlight exposure, and—when necessary—supplementation is crucial for providing a healthy metabolic environment for mothers and infants in utero. The Recommended Daily Allowance recommendations are shown in Table 2.

## 6. Interplay Connecting Magnesium and Vitamin D

### In-Depth Exploration of the Interconnected Physiological Process Influenced by Both Magnesium and Vitamin D

**Figure 2:**
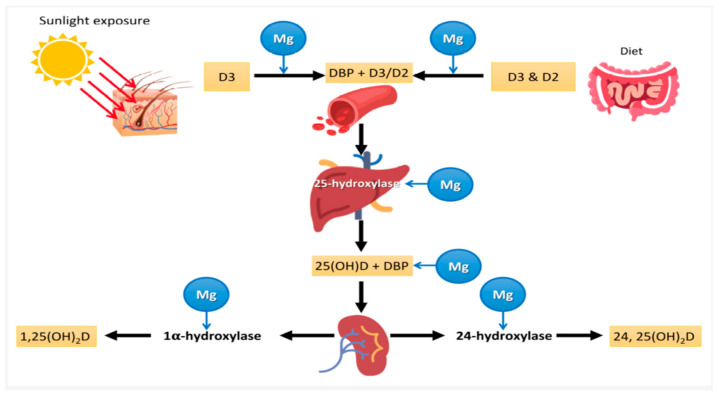
Vitamin D3 is created in the skin by a reaction between cholesterol and UVB radiation, which activates 7-dehydrocholesterol. Subsequently, either vitamin D3 or oral vitamin D (D2, ergocalciferol, or D3, cholecalciferol) undergoes conversion into 25(OH)D in the liver and further into the active hormonal metabolite 1,25(OH)_2_D (calcitriol) primarily in the kidneys, but also in other organs as required. The involvement of Mg is illustrated in this figure, highlighting its role as a cofactor essential for vitamin D binding to its transport protein, hepatic 25-hydroxylation of vitamin D, transport of 25(OH)D, and renal 1α-hydroxylation into its active hormonal form. Therefore, all these processes are dependent on magnesium. Picture adapted from [78].

Magnesium and vitamin D are interconnected in several physiological processes, and their deficiency may worsen each other’s impact. It is theorized that magnesium and vitamin D synergistically affect cardiometabolic health during pregnancy [77]. Understanding the intricate relationship between these two nutrients is crucial for developing comprehensive strategies to mitigate cardiometabolic risks. This interaction is shown in Figure 2 [78].

Magnesium and vitamin D are critical nutrients for the proper functioning of various organ systems. Magnesium aids in activating vitamin D, which is crucial for regulating calcium and phosphate homeostasis, thereby influencing bone growth and maintenance. All enzymes involved in vitamin D metabolism require magnesium, functioning as a cofactor in the enzymatic reactions within the liver and kidneys. The conversion of vitamin D from its inactive form (cholecalciferol or ergocalciferol) to its active form (calcitriol) relies on magnesium-dependent enzymes. These enzymes, including 25-hydroxylase and 1-alpha-hydroxylase, catalyze the transformation of vitamin D into 25-hydroxyvitamin D [25(OH)D] in the liver and subsequently into 1,25-dihydroxyvitamin D [1,25(OH)_2_D] in the kidneys [79]. Magnesium acts as a cofactor for enzymes responsible for vitamin D metabolism, with sufficient magnesium levels ensuring these enzymes function correctly, thereby increasing active vitamin D’s bioavailability [80]. Vitamin D enhances the intestinal absorption of calcium, while magnesium aids in transporting calcium across cell membranes. Both nutrients are crucial for supporting calcium homeostasis and bone health. A deficiency in either nutrient can impair calcium absorption and utilization, adversely affecting bone density and increasing the risk of osteoporosis [81,82]. Calcium is actively absorbed from the small intestine with the help of vitamin D. Together, calcium and phosphorus form hydroxyapatite crystals that mineralize and strengthen bones. Therefore, a diet rich in vitamin D and calcium is essential for proper bone mineralization [83]. Magnesium helps transport calcium across cell membranes; nutrients are crucial for maintaining calcium homeostasis and bone health [84]. Vitamin D deficiency can lead to decreased calcium absorption, and without adequate magnesium, the body may struggle to maintain proper calcium levels, affecting bone density and health.

Both nutrients regulate blood pressure, with vitamin D influencing the renin–angiotensin–aldosterone system (RAAS) to affect blood pressure and fluid balance. At the same time, magnesium helps relax blood vessels, thereby reducing blood pressure, and a deficiency in either nutrient can contribute to hypertension and increased cardiovascular risk.

The vitamin D receptor is expressed on various immune cells, such as B cells, T cells, and antigen-presenting cells, allowing these cells to synthesize the active vitamin D metabolite. This enables vitamin D to act in an autocrine manner within the local immune environment, modulating innate and adaptive immune responses. Deficiency in vitamin D is linked to higher autoimmunity and increased susceptibility to infection [85].

Magnesium is associated with nonspecific and specific immune responses and is crucial in various immune mechanisms. It aids in substance P binding to lymphoblasts, enhances T helper cell, B cell, and macrophage responses to lymphokines, and supports antibody-dependent cytolysis and immune cell adherence. Chronic magnesium deficiency leads to increased baseline inflammation associated with oxidative stress, which is related to various age-related morbidities [86].

Magnesium and vitamin D exhibit anti-inflammatory properties. Vitamin D has strong anti-inflammatory properties, helping to reduce pro-inflammatory mediators and increase anti-inflammatory cytokines [87]. Magnesium deficiency is linked to elevated C-reactive protein (CRP) and its precursors, such as interleukin (IL)-6 and IL-1, which are associated with the prevalence and severity of depression [88]. CRP is a commonly used marker of chronic systemic inflammation, and elevated CRP levels have been linked to depression [89].

Magnesium is needed for glucose metabolism, the synthesis of fats, proteins, nucleic acids, and coenzymes; muscle contraction; and methyl group transfer. Disruptions in magnesium metabolism can affect these functions [90]. Vitamin D controls the expression of genes related to energy production and mitochondrial function, thereby contributing to cellular energy metabolism [91].

Magnesium and vitamin D interaction is vital for multiple physiological functions, such as bone health, cardiovascular activity, immune function, and metabolic processes. Ensuring sufficient levels of both nutrients is critical for overall well-being, as deficiencies can change these linked systems in a domino effect. Supplementation can counteract these deficiencies.

Magnesium supplementation is commonly used to address deficiencies and support various aspects of health. Supplements come in various forms, including magnesium citrate, magnesium oxide, and magnesium glycinate, each with different levels of bioavailability and gastrointestinal tolerance [92]. Magnesium supplementation has been shown to benefit conditions such as muscle cramps [93], migraines [93], and cardiovascular health [94], and it may also help manage symptoms of stress and insomnia [95]. Recommended dosages vary depending on individual needs and health conditions, but typical daily doses range from 200 to 400 mg [96]. It is important to balance magnesium intake with other nutrients and monitor for potential side effects, such as diarrhea or abdominal discomfort.

Vitamin D supplementation is a widely used strategy to address and prevent vitamin D deficiency, particularly in individuals with limited sun exposure or dietary intake [15]. Supplementation is available in various forms, including vitamin D2 (ergocalciferol) and vitamin D3 (cholecalciferol). Vitamin D3 is generally preferred due to its superior efficacy in raising blood levels of vitamin D [97]. The recommended dosage varies depending on age, health status, and geographic location. Still, common guidelines suggest daily intakes ranging from 600 to 2000 IU for adults [98]. It is important to monitor blood levels and consult healthcare providers to avoid excessive intake, which can lead to toxicity [99].

## 7. Summary and Research Perspective

In summary, this review underscores the importance of ensuring optimal levels of magnesium and vitamin D during pregnancy for the health of mothers and infants. The evidence shows these nutrients’ significant impact on regulating blood pressure, glucose metabolism, and fetal development. Addressing deficiencies in magnesium and vitamin D is crucial for reducing the risk of cardiometabolic complications such as HDPs and GDM. Recognizing the interplay between these nutrients and their combined effect on maternal and fetal well-being highlights the urgent need for comprehensive approaches to promote proper nutrition during pregnancy. Further research and public health initiatives that advocate adequate magnesium and vitamin D intake are essential for enhancing pregnancy outcomes and long-term cardiovascular health for mothers and their children.

In conclusion, this narrative review highlights the critical roles of magnesium and vitamin D in maintaining maternal and fetal health during pregnancy. The evidence presented emphasizes the importance of adequate levels of these nutrients in regulating blood pressure, glucose metabolism, and fetal development. Addressing deficiencies in magnesium and vitamin D is crucial for mitigating cardiometabolic risks, including hypertensive disorders of pregnancy and gestational diabetes. Recognizing these nutrients’ interconnected nature and synergistic effects on maternal and fetal well-being underscores the need for comprehensive strategies to support optimal nutrition during pregnancy. The urgency of this issue is apparent, and there is an immediate need for future research and targeted interventions to alleviate the burden of pregnancy complications. This is a call to action for healthcare professionals, researchers, and policy makers in obstetrics and gynecology. Additional public health efforts are needed to promote adequate magnesium and vitamin D intake, which is essential for improving pregnancy outcomes and long-term cardiovascular health for both mothers and their offspring.

## Figures and Tables

**Table 1:** NIH guidelines for the Recommended Daily Allowance of Magnesium.

RDA Magnesium			
Age	Female	Pregnancy	Lactation
14–18 years	360 mg	400 mg	360 mg
19–30 years	310 mg	350 mg	310 mg
31–50 years	320 mg	360 mg	320 mg
51+ years	420 mg	320 mg	

**Table 2:** NIH Guidelines for Recommended Daily Allowance of vitamin D.

RDA Vitamin D			
Age	Female	Pregnancy	Lactation
14–18 years	15 mcg	15 mcg	15 mcg
	600 IU	600 IU	600 IU
19–50 years	15 mcg	15 mcg	15 mcg
	600 IU	600 IU	600 IU
51–70 years	15 mcg		
	600 IU		
